# Supplementary material for: Osteoarthritis in Pseudoxanthoma Elasticum Patients: An Explorative Imaging Study
Source: J Clin Med. 2020 Dec 1;9(12):3898. doi: 10.3390/jcm9123898 (PMC7760162; doi:10.3390/jcm9123898)
Supplement: Supplementary file 1 [file jcm-09-03898-s001.zip › jcm-980462-supp.docx]

**Supplementary**

**Table S1.** Number of patients with a missing per variable.

| **Variable** | **Number of patients with a missing (%)** |
| --- | --- |
| Age | 0 (0%) |
| BMI | 7 (4%) |
| Gender | 0 (0%) |
| Smoking status | 9 (5%) |
| AC-score | 2 (1%) |
| GH-score | 2 (1%) |
| Intervertebral disc score | 7 (4%) |
| Facet joint score | 5 (3%) |
| Hip score | 2 (1%) |
| Tibiofemoral score | 5 (3%) |
| Patellofemoral score | 5 (3%) |
| Ankle score | 11 (6%) |

The joint scores exist of several sub scores for osteophytes, marked joint space narrowing, and subchondral sclerosis/cysts or different levels in case of the facet joint and intervertebral disc score. If one of the subscores was missing, it was counted as a missing patient. *AC Acromioclavicular; GH glenohumeral*

**Table s2.** Results from the subgroup analysis within the PXE patients.

| **Joint score (Number of truncated mutations)** | **Crude OR (95% CI)** | ***p*-value** | **Adjusted OR (95% CI)** | ***p*-value** |
| --- | --- | --- | --- | --- |
| AC score (0 vs 2) | 0.216 (0.056–0.828) | 0.025 | 0.154 (0.033–0.712) | 0.017 |
| AC score (1 vs 2) | 0.616 (0.348–1.092) | 0.097 | 0.694 (0.372–1.296) | 0.252 |
| Tibiofemoral score (0 vs 2) | 0.420 (0.128–1.375) | 0.152 | 0.472 (0.119–1.866) | 0.284 |
| Tibiofemoral score (1 vs 2) | 0.383 (0.207–0.707) | 0.002 | 0.407 (0.208–0.797) | 0.009 |
| Patellofemoral score (0 vs 2) | 0.154 (0.032–0.745) | 0.020 | 0.137 (0.025–0.739) | 0.021 |
| Patellofemoral score (1 vs 2) | 0.806 (0.451–1.440) | 0.466 | 0.971 ( 0.522–1.804) | 0.925 |

Table 2. truncating; 1 truncated and 1 non-truncated; 2 non-truncating gene variants. To test whether the prevalence of AC, tibiofemoral and patellofemoral OA differed between these groups, ordinal logistic regression models were built adjusting for age, body mass index (BMI) and smoking status. The group of patients with 2 truncating *ABCC6* variants was used as a reference category.
